# Using unmanned aerial systems and deep learning for agriculture mapping in Dubai

**DOI:** 10.1016/j.heliyon.2021.e08154

**Published:** 2021-10-11

**Authors:** Lala El Hoummaidi, Abdelkader Larabi, Khan Alam

**Affiliations:** a Laboratory Analysis and Modeling of Water and Natural Resources (LAMERN), Mohammed V University in Rabat, Mohammadia School of Engineers, Rabat, Morocco; b Geographic Information Systems Centre (GISC), Dubai Municipality, Dubai, United Arab Emirates; c Department of Physics, University of Peshawar, Khyber Pakhtunkhwa, Pakistan

**Keywords:** Agriculture mapping, Deep learning, GIS, Drone mapping, Precision agriculture, Multispectral images, UAV, UAS

## Abstract

As part of the sustainable future vision, sustainable agriculture has become an essential pillar of the food security strategies formulated by the Dubai Government. Therefore, the Dubai Emirate began relying on new technology to increase productivity and efficiency. Agriculture applications also depend on accurate land monitoring for timely food security control and support actions. However, traditional monitoring requires field surveys to be performed by experts, which is costly, slow, and rare. Agriculture monitoring systems must be furnished with sustainable land use monitoring solutions, starting with remote sensing using drone surveys for affordable, efficient, and time-sensitive agriculture mapping. Hence, the Dubai Municipality is currently using Unmanned Aerial Vehicles (UAVs) to map the farming areas all over the Emirate, support locating lands conducive to cultivation, and create an accurate agriculture database contributing to the decision-making process in determining areas suitable for crop growth. This study used a novel object detection method coupled with geospatial analysis as an integrated workflow to detect individual crops. The UAV flights were executed using a Trimble UX5 (HP) over twelve communities across the Dubai Emirate for six months. Detection methods were applied to high-resolution drone images, consisting of RGB and near-infrared (NIR) bands. Advanced geoprocessing tools were also used to analyze, evaluate, and enhance the results. The performance of detection of the selected deep learning models are discussed (vegetation cover accuracy = 85.4%, F1-scores for date palms and ghaf trees = 96.03% and 94.54% respectively, with respect to visual interpretation ground truth); moreover, sample images from the datasets are used for demonstrations. The main aim is to offer specialists a solution for measuring and assessing living green vegetation cover derived from the processed images that is integrated. The results provide insight into using UAS and deep learning algorithms as a solution for sustainable agricultural mapping on a large scale.

## 1 Introduction

Agriculture is, without a doubt, one of the most significant factors in the sustainability of any economy [1, 2]. It plays a key role in long-term economic growth and structural transformation, though it may vary considerably by country [3, 4, 5, 6]. In the past, agricultural activities were limited to food and crop production. Nonetheless, they have evolved in several countries to the processing, marketing, and distribution of crops and livestock products. Currently, agricultural activities constitute the first source of livelihood, improving GDP [7], being a source of national trade, reducing unemployment, providing raw materials for production in other industries, and overall developing the economy [8, 9, 10].

Remote sensing is used extensively by governments and the private sector to map soil properties, the classification of crop types, the detection of crop water stress, the monitoring of crop diseases, and the mapping of crop yield [11, 12]. Such technology, which involves using sensors coupled with geospatial analysis tools, brings data from multiple sources to support decisions associated with crops. Data can be captured through Unmanned Aerial Systems (UAS), known as drones, with a more flexible spatial and spectral resolution compared with other remote sensing platforms [13]. Furthermore, land cover classification based on remote sensing imagery has been used in change detection monitoring, agricultural management, green vegetation classification, biodiversity conservation, land use, and urban planning [14, 15]. One of the most significant applications of land cover classification is vegetation detection. Hence, several options for generating land cover maps were investigated by researchers and experts, including digital photo interpretation, supervised and unsupervised classification, classification and regression trees (CART), and deep learning object detection [16, 17, 18, 19, 20].

Since 2012, deep learning methods have been used extensively for land cover classification, especially after several advances were reported in various computer vision tasks, including image classification, object detection, tracking, and semantic segmentation. Snehal et al. [21] used convolutional networks for multispectral image classification. Zhang et al. [22] elaborated a great review of object detection approaches to land cover classification using high-resolution multispectral imagery. The authors evaluated and tested the performances of several deep learning models against the traditional methods and concluded that the deep learning-based approaches provide an end-to-end solution and demonstrate better performance than the conventional, pixel-based methods by utilizing both spatial and spectral information. Several other works have also demonstrated that artificial intelligence and deep learning methods are reasonably promising for land cover classification and vegetation detection in particular [23, 24, 25].

With world commodity prices at an all-time low and supply at an all-time high due to increasing food consumption and production demands, the modern farming industry is at a turning point [26]. There is a more noticeable need than ever before for agronomists and farmers across the globe to improve resource management in response to tight and fragile budgets and increasing pressure for enhanced product quality. The Dubai Emirate is no different. Thus, while Dubai is working to make the most of its re-export hub and global gateway status in the fresh food sales sector, which is currently estimated at about 280.5 million tons in volume according to a report by the Emirates Authority for Standardization and Metrology (ESMA), there was a 53% increase in organic farms across Dubai in 2019. The report also notes an 89% increase in production from 1,240 tons in the last quarter of 2018 to 2,356 tons in the first quarter of 2019. Therefore, the Dubai Municipality has introduced new projects to survey and map agricultural areas using drones and connected analytics that can support an automated workflow to assess crop health, make informed decisions with plant count, access actionable real-time quality data, assess agriculture-related damage, and prepare plans to mitigate losses after the spread of a particular disease or an extreme weather event. These projects have enabled the Dubai Municipality to provide growers, service providers, and agriculture researchers with a quick and effective way to scout their crops, identify stress, create treatment plans, track plant growth, and much more through the utilization of high-resolution multispectral drones that can detect and quantify crop health problems. Such valuable insights support the reduction of input costs and boost yield. Furthermore, the multispectral drone data reveals field variability invisible to the naked eye, which helps catch diseases early, allows for a timely response, and improves yields.

Throughout this paper, we evaluate the suitability of UAS-based remote sensing to monitor crops in Dubai using a novel object-based vegetation detection method, which utilizes the Normalized Difference Vegetation Index (NDVI) and deep learning techniques. This paper has the following contributions:

− Introduction of a novel object-based vegetation and tree detection method that utilizes NDVI and deep learning techniques, yet the method is simple; it outperforms the two other investigated methods (supervised classification and photo interpretation) in performance and accuracy detection.
− Demonstrating the potential use of an NDVI image as a replacement to a standard RGB to get improved results.
− Discussion of the underlying reasons why our deep learning model could perform better than other methods and potential strategies to use deep learning in many further applications.

## 2 Materials and methods

### 2.1 Study area

**Figure 1:**
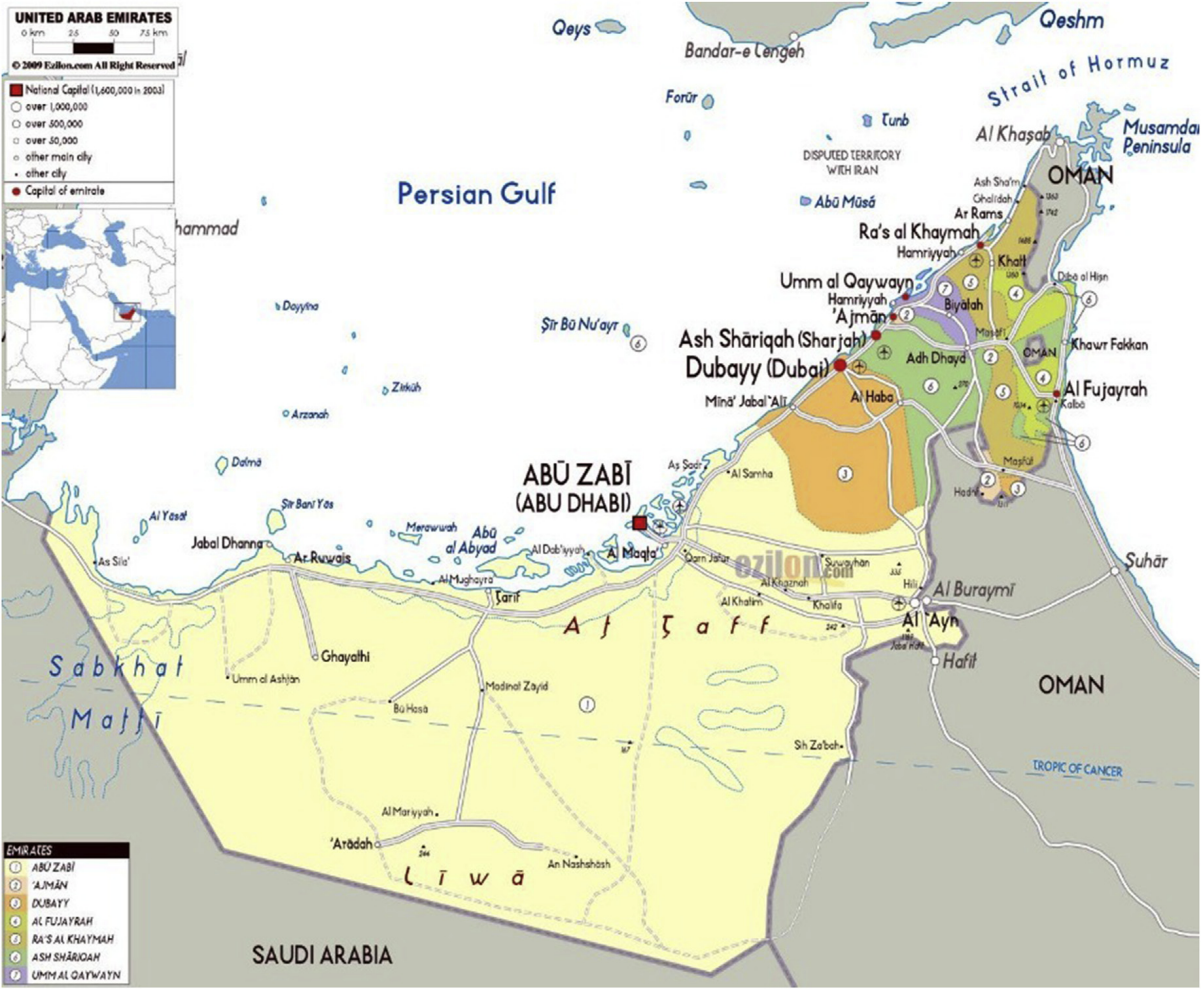
Location Map of Dubai Emirate and major cities in the United Arab Emirates.

The Dubai Emirate is the second largest of the seven emirates that constitute the United Arab Emirates. Positioned on the southeast coast of the Arabian Gulf between 55°18′East and 25°16′North, Dubai has a total area of 3,900 square kilometers and stretches along the Arabian Gulf coast for 72 km. The Emirate of Dubai shares borders with Sharjah in the northeast, the capital Abu Dhabi in the south, and the Sultanate of Oman in the southeast. Figure 1 shows the administrative boundaries of the Dubai Emirate, which accounts for 5% of the United Arab Emirates total area.

Dubai's landscape is a combination of shallow shores, sandy desert, and coral reefs. Dubai's waters are the living environment of more than 300 species of fish, and such rich marine life has provided Dubai's inhabitants with a significant source of income for thousands of years. Most of Dubai's cultivated land consists of date palms and is cultivated in the arc of small oases that constitute the Hatta Area. The Dubai Municipality provides incentives to farmers. For example, it offers a 50% subsidy on fertilizers, seeds, and pesticides. It also provides loans for machinery and technical assistance [27, 28].

Table 1 demonstrates that 13% of cultivated land in the Dubai Emirate is used to grow vegetables, 32% fruit, 14% feed crops, and 43% for other uses. The most productive region is Hatta, which has underground water supplies from the nearby mountains of Oman, which enjoys high rainfall. The main crops are tomatoes, melons, and dates. At the same time, vegetable growers in Dubai overcame the desert challenges, and more than 27 tons were produced in 2019, as shown in Table 2.

**Table 1:** Distribution of Land Use (Area in Donums-dūnum) - Emirate of Dubai (2017–2019).

Years	Fruit Trees (in dūnum)	Feed Crops (in dūnum)	Vegetables (in dūnum)	Forest Trees (in dūnum)	Temporary Fallow (in dūnum)	Other Lands (in dūnum)	Total (in dūnum)
2017	15,810	3,177	1,765	760	1,725	21,645	44,882
2018	15,903	3,218	1,660	784	1,664	18,464	41,693
2019	20,409	8,108	7,529	784	1,917	23,168	61,914
Area %	32%	14%	13%	1%	3%	37%	100%

**Table 2:** *Vegetables by Crop - Emirate of Dubai (2019)*.

Crop	Value (in 000 AED)	Average of production (in Tons/Dūnum)	Quantity (in Tons)	Area (in Dūnum)
Tomatoes	18,030.9	6.2	6,297.9	1,010.8
Cucumber	8,166.9	9.4	2,946.7	313.9
Pepper	1,637.1	4.9	403.3	82.5
Squash	8,517.3	2.3	2,793.9	1,219.8
Eggplants	4,575.6	3.3	2,333.8	716.6
Cauliflower	7,216.0	3.5	2,590.3	740.1
Cabbage	7,488.8	6.0	4,082.0	680.3
Watermelon	474.9	3.0	391.9	130.3
Leafy Vegetables	1,740.7	1.3	579.9	447.1
Other	10,567.6	2.3	4,970.5	2,187.3
**Total**	**68,415.8**	**3.6**	**27,390.3**	**7,528.8**

The major vegetable crops supplying most of the Emirate's needs during the season are tomato, cabbage, eggplant, squash, and cauliflower. In addition to dates, the key fruit crops are citrus and mangoes [29], as shown in Table 3.

**Table 3:** *Fruit Trees by Crop - Emirate of Dubai (2019)*.

Crop	Value (in 000 AED)	Average of production (in Tons/Dūnum)	Quantity (in Tons)	Area (in Donum)
Palm Tree	97,034.9	0.8	15,172.4	19,124.9
Lime	1,623.0	3.2	516.0	161.0
Lemon Adalia	294.0	3.2	93.0	29.0
Grapefruit	146.0	2.4	59.0	25.0
Other Citrus	768.0	2.0	260.0	130.0
Mango	520.0	2.4	94.0	39.5
Guava	388.0	2.4	90.0	38.0
Chico	1,882.0	1.0	538.0	538.0
Lotus Jujube	1,824.0	3.2	608.0	190.0
Pomegranate	91.0	1.6	16.0	9.9
Fig	1,097.0	1.0	58.0	57.8
Almond	499.0	2.4	125.0	51.8
Other	129.0	2.5	35.0	14.0
**Total**	**106,295.9**	**0.9**	**17,664.4**	**20,408.9**

While obstacles such as severe environmental conditions, scarcity of water resources, and soil salinity have affected development, Dubai has engineered innovative solutions to overcome these challenges. These solutions are widely connected with underground aquifers or underground water supplies from the mountains. According to Table 4, agriculture in Dubai is carried out on a total, cultivable area of around 8,000 dunums, most of which are taken up by Rhode grass and alfalfa.

**Table 4:** *Field Crops by Crop - Emirate of Dubai (2019)*.

Crop	Value (in 000 AED)	Average of production (in Tons/Dūnum)	Quantity (in Tons)	Area (in Dūnum)
Alfalfa	20,056.8	6.0	12,535.5	2,089.2
Rhode grass	32,362.3	6.0	21,574.9	3,595.8
Sorghum	11,953.0	6.0	7,470.6	1,245.1
Maize	2,059.4	2.8	2,059.4	748.9
Other	3,343.1	6.0	2,571.6	428.6
**Total**	**69,774.6**	**5.7**	**46,212.0**	**8,107.6**

### 2.2 Overall study workflow

**Figure 2:**
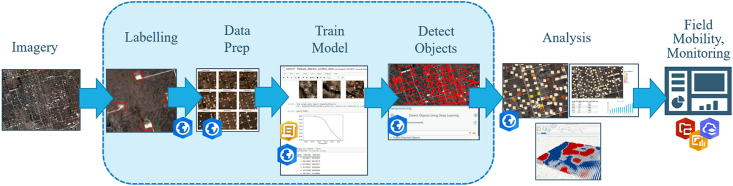
End-to-end from raw imagery to structured information about vegetation cover feature layers.

The workflow as described in Figure 2 shows the steps followed, namely acquiring high resolution, multispectral imagery, labeling, preparing the input datasets, training the models, detecting the objects, analyzing the results, and validating the outputs using field mobility monitoring through field visits. In this study, the exported training data and the training of the deep learning models that were used across the area of interest were done using the ArcGIS API for Python.

The deep learning workflows discussed later in this section had been enhanced for deploying trained models for tree feature extraction and vegetation cover classification, while PyTorch and fast.ai deep learning libraries were used for the data preparation, augmentation, and model training workflows. The object detection models used accepted training samples in the PASCAL_VOC_rectangle (pattern analysis, computational learning, statistical modeling, and visual object classes) format. For instance, the PASCAL VOC dataset is considered a standardized image dataset for object class recognition. On the other hand, the created label files are in XML format containing detailed information about image name, class value, and bounding boxes.

Once the training samples were exported, we fed them into the model for training. Data preparation was a time-consuming process involving massaging and collating the training labels into the specific format needed by each deep learning model. Typical data processing pipelines involved splitting the data into validation and training sets, applying multiple data augmentation techniques, creating the required data structures for loading data into the model, and setting the appropriate batch size. In our study, we automated all these time-consuming tasks using ArcGIS Pro. We could directly read the training samples exported by ArcGIS and construct the appropriate fast.ai DataBunch from it. This DataBunch consisted of training and validation DataLoaders with the specified transformations for data augmentation, chip size, batch size, and split percentage for the train validation split.

### 2.3 Drone data

**Figure 3:**
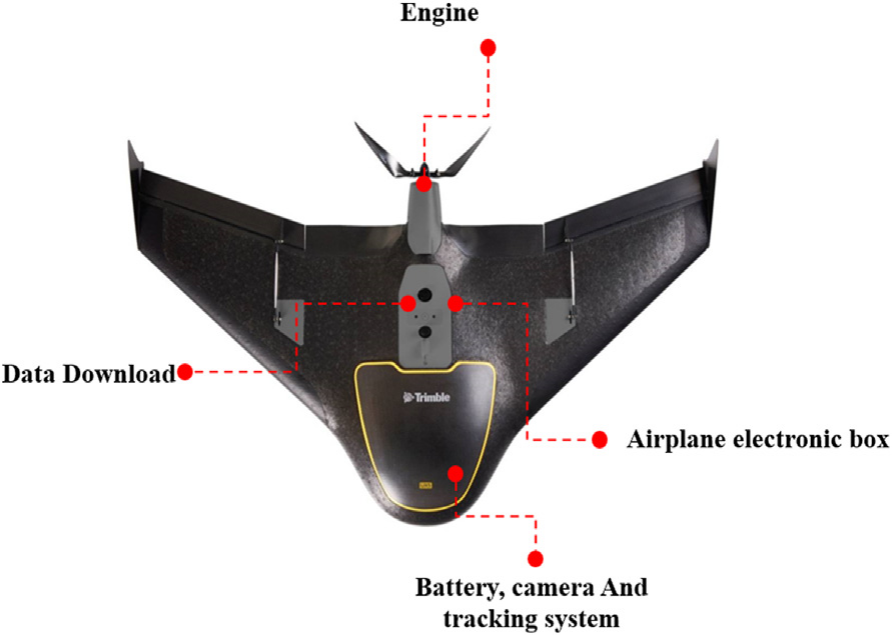
Trimble Drone used for data collection.

Figure 3 describes the different components of the Trimble UX5 HP UAS as the primary device used for capturing field data. It is an easy-to-use, fully automated, high resolution device capable of capturing aerial photography with resolutions down to one centimeter. It also provides an intuitive workflow that quickly creates the highest quality orthomosaics and 3D models for agriculture mapping, field leveling, progress monitoring, and asset mapping [30].

The multispectral drone imagery captured by the field team helps generate NDVI maps that can segregate soil from forest or grass, differentiate between crops at different crop stages, and detect plants that are under stress. Strong correlations have been proven between NDVI data measured at certain crop stages and crop yield. Hence, tracking crop growth at crucial stages helps provide an accurate estimate of the crop yield and addresses issues early [31, 32]. Table 5 lists the different levels of acquisition performance of the Trimble UX5 Drone.

**Table 5:** *Acquisition Performance (Trimble UX5 Drone)*.

Resolution (GSD)	1 cm–25 cm (4–99 in)
Height above take-off location (AGL)	75 m–750 m (246–2,460 feet)
Absolute accuracy XY/Z (no ground control points)	down to 2–5 cm
Relative orthomosaic/3D model accuracy	(1–2x/1–5x GSD)
Resolution (GSD)	1 cm–25 cm (4–99 in)

**Figure 4:**
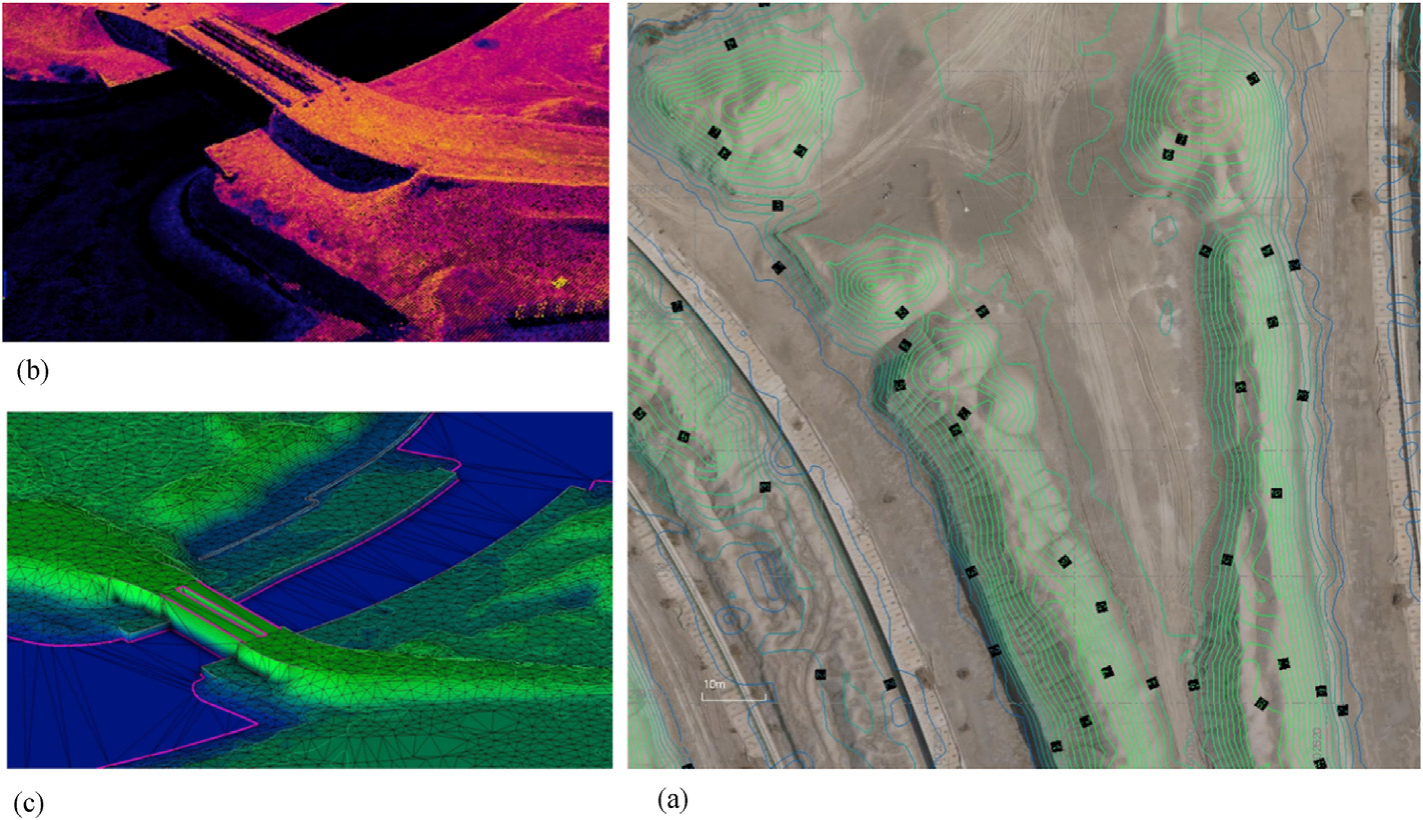
Drone Mapping Outputs (a: Corrected image showing contours, b: Three-dimensional surface built from point clouds, c: three-dimensional surface built from contour lines for the same area).

The drone used by the Dubai Municipality is equipped with photogrammetric and navigation equipment with a ground resolution of up to three centimeters. It is programmed to detect details such as NDVI, water stress, and a lack of specific nutrients in crops. The drone-supporting mapping efforts of the GISC are now being mainstreamed under the Emirate's disaster risk reduction and management (DRRM) and climate change adaptation (CCA) strategies. The Trimble UX5 HP drone used during the field survey is equipped with a modified color-infrared (CIR) Sony NEX5R fitted with a 16 mm lens. On each flight day, roughly 100 ground-based NDVI measurements were collected with the Trimble Green Seeker Handheld at a constant height of 80 cm above the target, of which the center point was georeferenced to two centimeters accuracy using a Trimble R8RTK GNSS system [33]. Figure 4 shows examples of the drone output such as an orthorectified image with elevation contours, the three-dimensional surface which is processed using the collected point clouds, and the generated three-dimensional surface using the processed contour lines.

The aerial survey mission is highly critical as the drone is flying in real airspace along with civil aircraft, commercial flights, VIP helicopters, private helicopters, etc. Before planning any mission, some of the most essential requirements are to provide proof of safe operation, ultimate coordination, and clear communication between the flight operators. In the Dubai Municipality, the first step consists of preparing a flight plan showing the range, mission type, overlap setting, and predicted operating location. After this, based on the plan and the geographical boundaries of the area to cover, an estimate of the adequate number of Ground Control Points (GCP) required is calculated. Finally, a detailed risk assessment document is elaborated along with a safety case plan which is shared with the drone operators.

**Figure 5:**
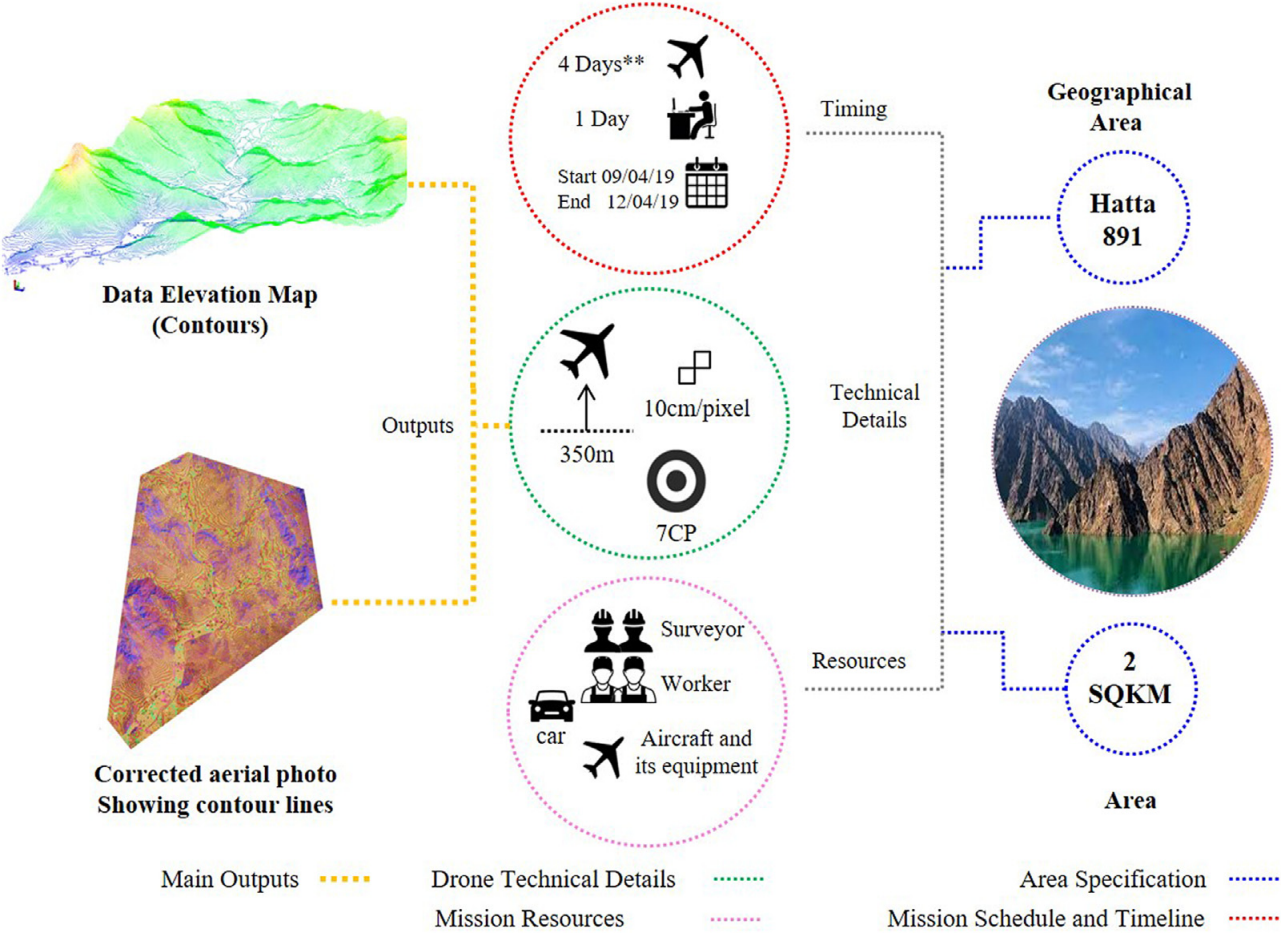
Process of drone mapping field mission – Hatta region.

Figure 5 summarizes the process of capturing aerial data with downward-facing multispectral cameras and LIDAR payloads using a Trimble UX5. During such a survey, the ground is photographed several times from different angles, and each image is tagged with coordinates. The field survey takes four days to cover the entire Hatta site (two square kilometers). In contrast, it takes only one day only for the Data Elevation Model (DEM) and rectified orthophoto of the area to be automatically produced.

To cover the twelve areas in the scope of this study with a total area of 770 square kilometers, five teams of four were dedicated to coordinate the flying process throughout the year, and with proper planning, all missions were successfully completed within 139 days, while the processing took around 78 days overall. Some areas were more accessible than others, such as AL WOHOOCH and SAIH SHUAIB, due to the absence of variety in surface features and the dominance of flat sand dunes. Table 6 elaborates, in detail, the flying and processing time in days for each community, along with the number of flights and number of repetitions during 2019.

**Table 6:** *UAS missions' details for areas scope of the study*.

Community	Sq. km	Flying time in days	Processing time in days	Number of flights	Repetitions per year (2019)
SAIH SHUAIB	41.61	5	4	5	1
HADAEQ SHEIKH MOHAMMED BIN RASHID	38.68	19	3	38	2
ALEYAS	10.52	5	8	5	1
AL KHEERAN	7.33	4	6	12	3
AL LESAILY	112.69	13	10	13	1
MARGHAM	152.59	25	12	25	1
AL WOHOOSH	26.51	3	2	3	1
AL MAHA	41.73	21	4	42	2
REMAH	82.87	5	7	5	1
GRAYTEESAH	91.83	12	8	12	1
AL FAGAA	140.53	15	13	15	1
HESSYAN	23.85	12	1	12	1
**Total**	**770.75**	**139**	**78**	**187**	

**Figure 6:**
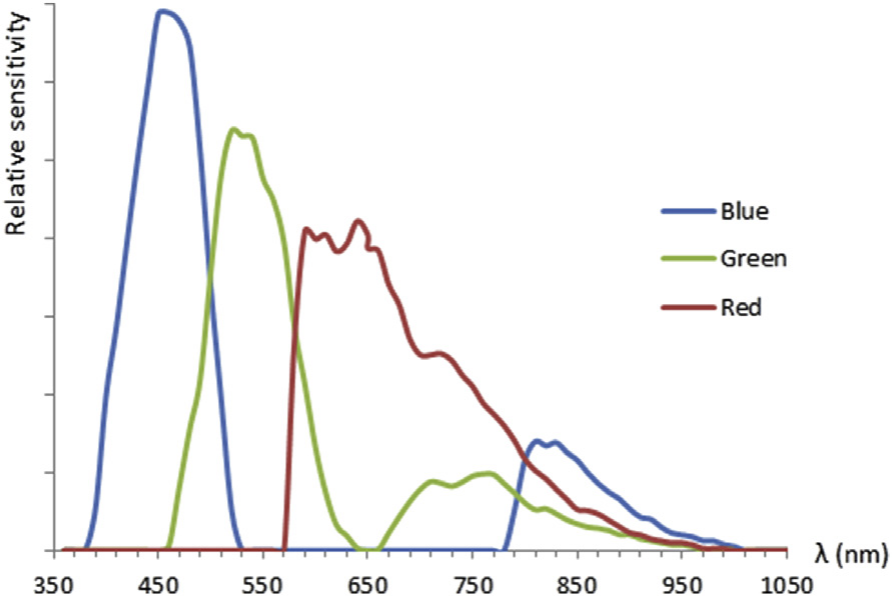
Spectral response of the Trimble UX5 HP Sony NEX-5N.

The Sony NEX5R was modified to get 3-band R-G-NIR imagery. In this configuration, pixels covered by a blue filter receive only NIR, while pixels covered by green and red filters receive mostly visible green or red, together with some of the red edge and NIR. The camera was set to simultaneously store 16.1 MP 3-band 14 bit per band linear lossy compressed RAW files (35 MB each, in Sony's proprietary ARW format) with 16.1 MP 3-band 8 bit per band gamma-compressed JPEG files, roughly 15 MB each [34]. Figure 6 demonstrates the true color (RGB) spectral responses of the Trimble UX5 device.

### 2.4 Processing and analysis

The processing methodology developed during this study comprised four steps: photogrammetric pre-processing, object detection using deep learning algorithms, data analysis, and evaluation of results. The first step was about pre-processing the UAS derived imagery using digital photogrammetry. Second, deep learning algorithms were selected and executed to detect the vegetation cover and identify related diseases, then template matching was performed to segment the main crop covered area and detect individual crops, respectively, on the orthophoto mosaic. Third, data analysis was performed using advanced geoprocessing tools. Finally, the detection accuracy threshold was determined, and a comparison between crop volume, crop pest estimates, and field samples was undertaken [35].

#### 2.4.1 Pre-processing

We imported all data layers into ESRI ArcGIS for Desktop 10.5, and pixel-level alignment of all data layers, including ground measurements, was ensured before analysis. The resulting orthomosaics were initially shifted in relation to the UX5 HP orthomosaics and ground measurement data. Therefore, prior to analysis, an automatic registration to the UX5 HP data using a second-order transformation was applied to the resultant orthomosaics in ArcGIS Pro 2.4. To analyze the reliability of each data type in multitemporal monitoring, UAS-based NDVI values and spectral profiles for three vegetation types (palms, ghafs, and grassland) were plotted over time for the available data acquisitions. The raster datasets were converted to point features using an Extract, Transform and Load (ETL) script, of which 0.1% was randomly selected before interpolation. The natural neighbor interpolation technique was selected as it is appropriate for point features with an irregular distribution and density; it limits overshoots of local high values and undershoots of local low values. The digital terrain model was generated from the digital surface model to calculate the crop height model.

**Figure 7:**
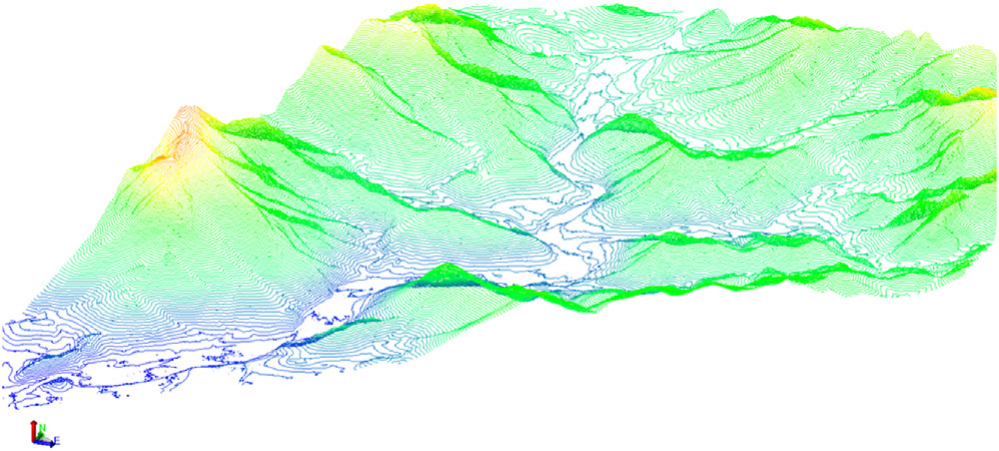
Generated contour map- Hatta region.

Topographic maps are critical for agriculture and vegetation mapping [36]. For example, some vegetation species may only grow in areas with an elevation higher than a certain level. Therefore, it is essential to use drones to capture data as aligned by aerial triangulation, then orthorectify and geo-reference it using the GCP information. Figure 7 shows the final DEM and contour lines generated for the Hatta region from orthorectified drone imagery.

#### 2.4.2 Object detection algorithms (deep learning)

**Figure 8:**
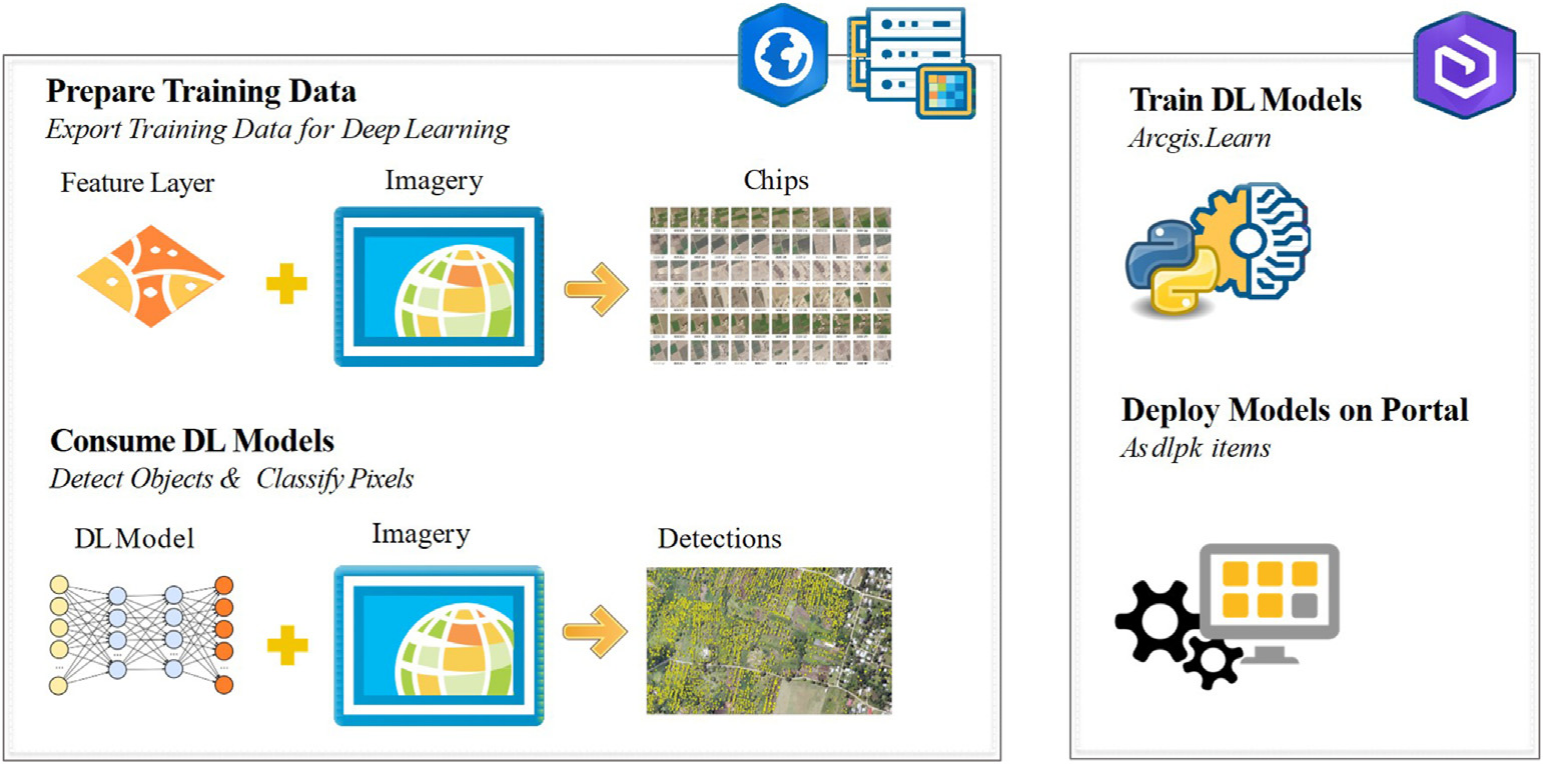
General Deep Learning Packages (DLPKs) usage workflow.

With the existing capabilities in ArcGIS, the model of classifying points representing trees in point cloud datasets was evaluated along with over a dozen deep learning models on geospatial datasets. However, the models selected and then enhanced during this study were "the tree Point Classification model" and "the Landcover Classification model." Both these models were modified using Python scripts to adapt them to the arid nature of Dubai, then were successfully processed to detect the classified vegetation cover and individual trees from the corrected input images through integration with TensorFlow, a third-party training software. Figure 8 summarizes the overall workflow from preparing the training data to consuming the deep learning models. It also shows how the ArcGIS.learn module in the ArcGIS API for Python is used to train deep learning models, which is an iterative process and can enhance the outputs significantly. The final models are deployed as deep learning packages (DLPKs) and are archived, documented, and shared with the project team. All the algorithms and tools described in the paper were implemented using the Python programming language. All experiments were run in a workstation using a Windows Server 2012 R2 operating system with 16 dual-core 3GHz processors and an NVIDIA GPU graphics board.

**Figure 9:**
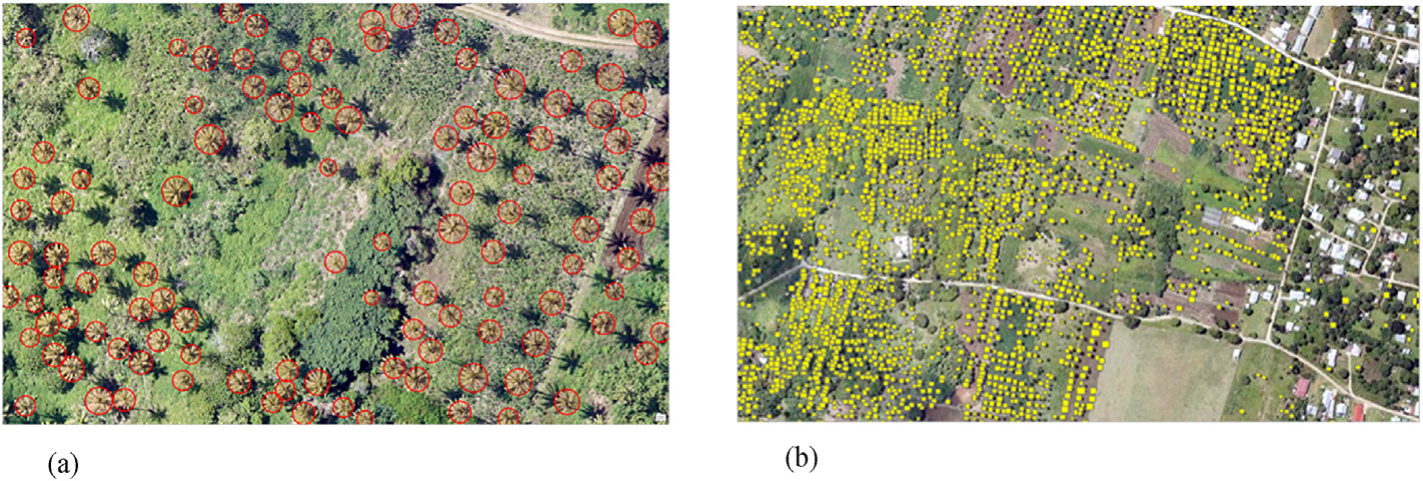
a: Training samples recorded for the palm trees, b: Generated results for a bigger area through object detection algorithm.

The most onerous tasks of the work to extract features from the imagery were preparing the data, creating training samples, and training the model. At this stage of the process, these steps have been completed, and a trained model to detect different types of crops is used throughout the processed drone imagery. Object detection requires multiple tests to achieve the best results. We adjusted many parameters to ensure the model performed at its best. To test these parameters, the detection was tested in a small section of the image until the results were satisfactory, then the detection tools were extended to the covered areas by the drones [37]. Figure 9 demonstrates some of the training samples labeled for the palm trees, along with the generated results of the object detection algorithm used during this study.

**Figure 10:**
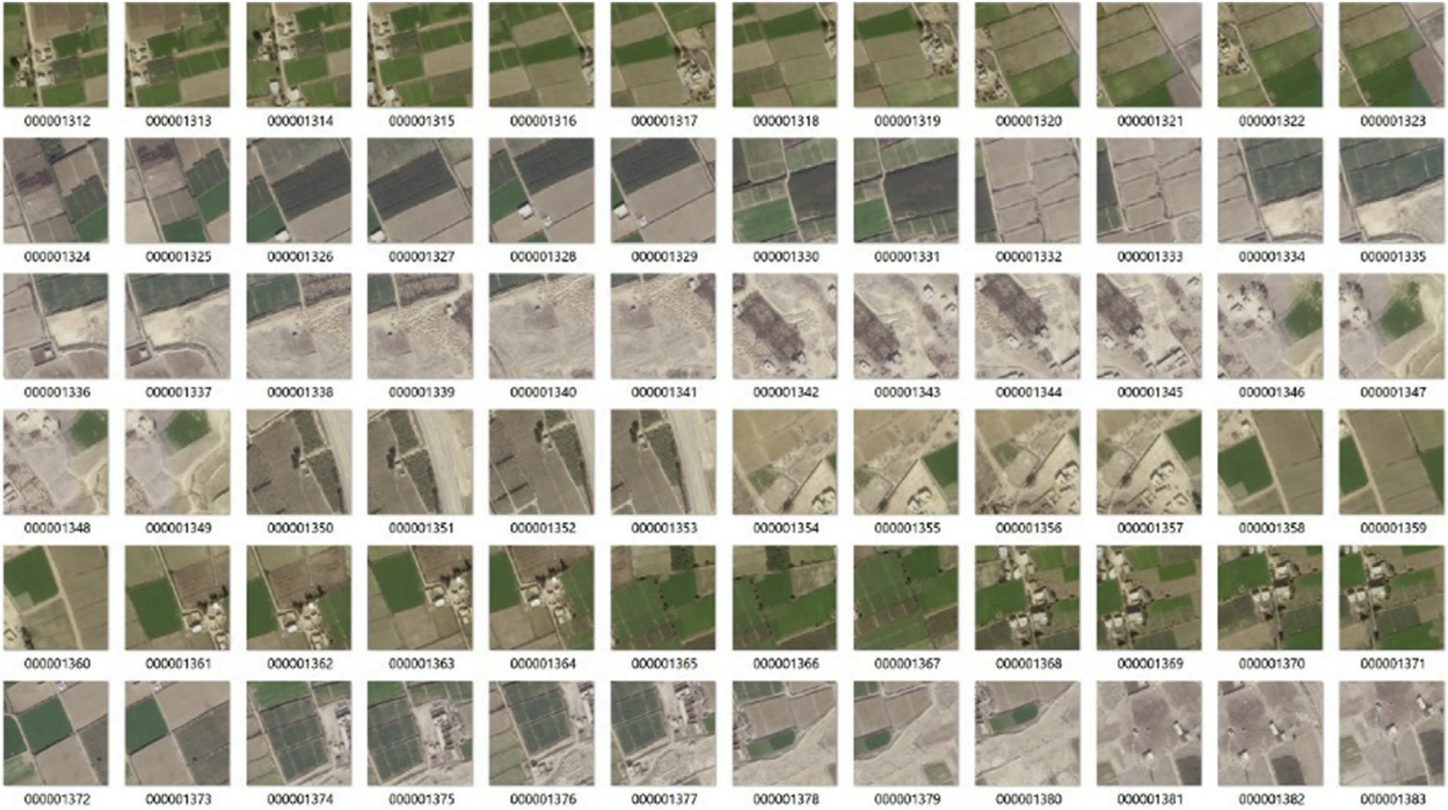
Training samples recorded for field crops.

When training deep learning or image classification models, creating good training samples is critical. It is also often the most challenging and time-consuming step in the process. To provide our deep learning model with the information it needed to extract all the crop types in the image, we created features for several palm trees and other field crops to teach the model what size, shape, and spectral signature these objects may have. The deep learning class training samples are based on small sub-images, called image chips, containing the feature or class of interest. Figure 10 shows a subset of the image chips used for this study [38, 39].

**Figure 11:**
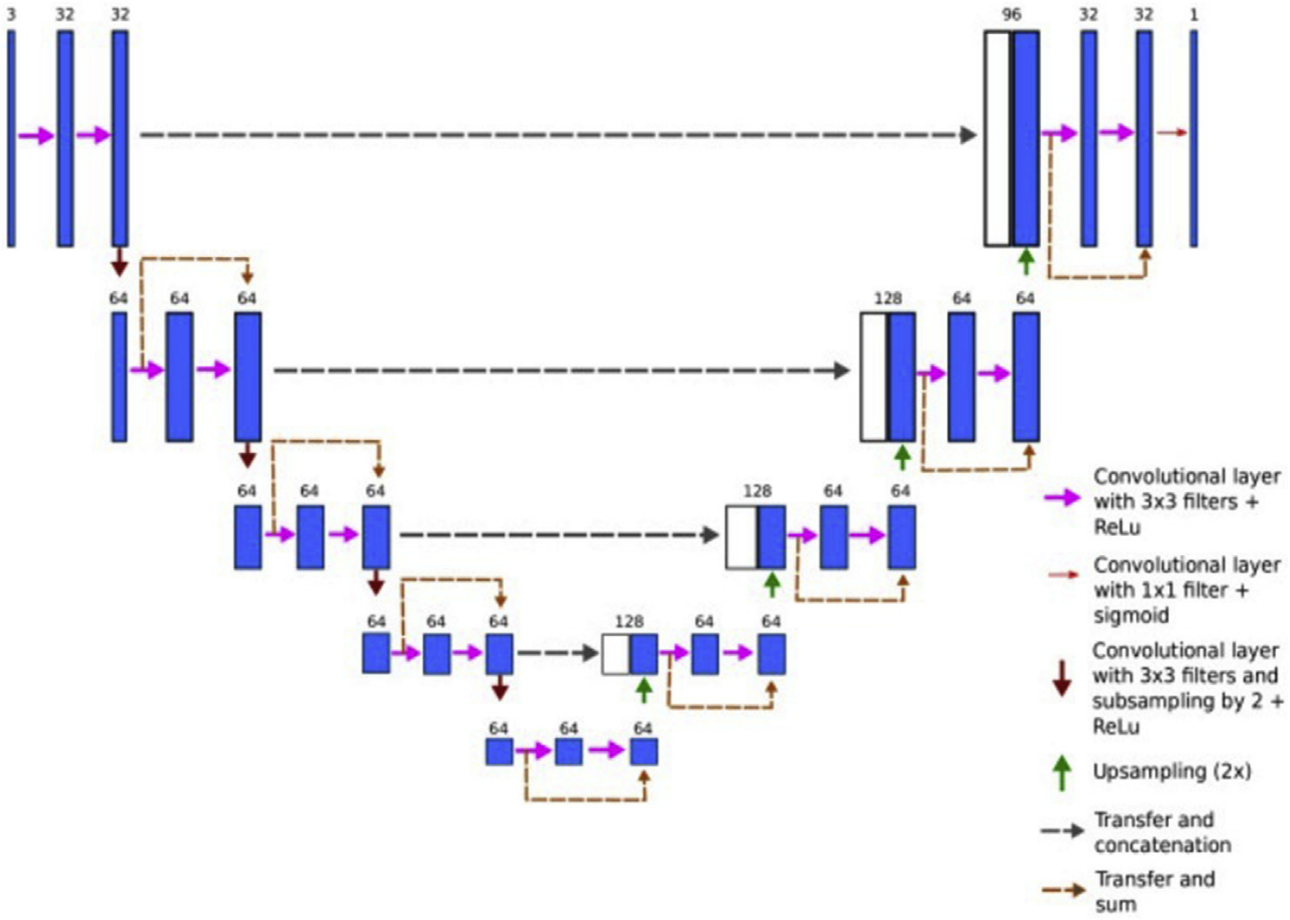
U-net architecture. Blue boxes represent multi-channel feature maps, while white boxes represent copied feature maps. The colored arrows represent different operations.

The ArcGIS.learn module in ArcGIS API for Python was used to apply our deep learning model in the workflows selected for this study (U-net) [40, 41]. Its architecture can be considered as an encoder network followed by a decoder network. Unlike the concept of classification, where the result of the deep network is the only important element, semantic segmentation requires discrimination at pixel level along with a protocol to project the detected features that were learned at different stages of the encoder onto the assigned pixel space. The encoder is the first part of the architecture diagram. It is a pre-trained classification network like VGG/ResNet where convolution blocks are applied, followed by downsampling (maxpool) to encode the input image into feature representations at different levels. As shown in Figure 11, the decoder is the second part of the architecture. The main goal is to semantically project the detected features at a lower resolution as learned by the encoder onto the pixel space at a higher resolution to get the best dense classification. The decoder focuses on regular convolution operations after processing the upsampling and concatenation functions.

**Figure 12:**
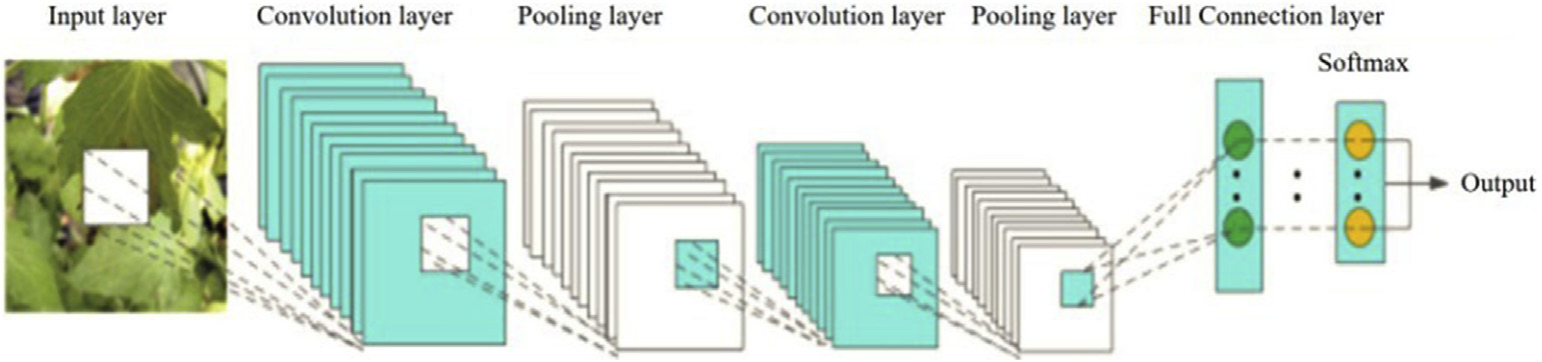
*CNN Network layers*.

In computer vision tasks, tree detection tends to be the concept of human experience rather than a purely mathematical definition [42]. Compared with other image recognition methods, object detection based on deep learning doesn't go through feature extraction first, rather it goes through iterative learning that can find appropriate features that acquire contextual and global features of images and are more robust with higher recognition accuracy. In this study, Convolutional Neural Networks (CNN) as a complex network structure is used to extract such information from the processed high resolution imagery. As shown in Figure 12, the CNN model is composed of an input layer, a convolution layer, a pooling layer, a full connection layer, and an output layer. In our model consumed by ArcGIS Pro, the pooling and convolution layers alternate several times. When the neurons of the pooling layer are connected to the neurons of the convolution layer, no full connection is required. In the real natural environment, the significant differences in texture, shape, color, size, background, and imaging reflectance of plant diseases and pests make this stage of recognition a challenging task. Thanks to CNN's advanced feature extraction capability, choosing a CNN-based classification network over other options has become the most common pattern vegetation cover detection method [43].

#### 2.4.3 Data analysis

This study's primary analysis focuses on estimating vegetation health, as extracted from the same images used for deep learning extraction by calculating a vegetation health index. To assess vegetation health, the Visible Atmospherically Resistant Index (VARI) should be calculated [44], which was designed as an indirect measure of leaf area index (LAI) and vegetation fraction (VF) using only reflectance values from the visible wavelength:

(1) (Rg−Rr)/(Rg+Rr−R(Rg−Rb))

**Figure 13:**
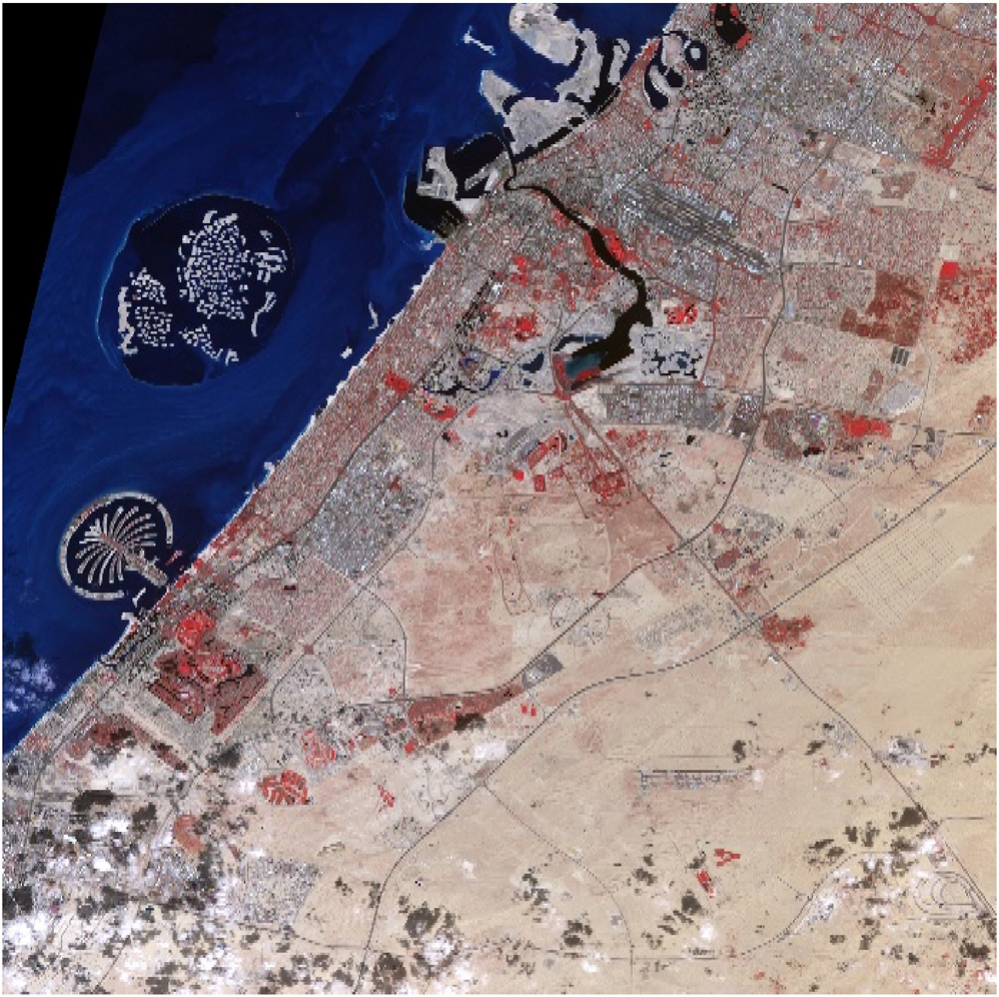
NDVI results generated from drone imagery (HESSYAN and JABAL ALI communities).

Where Rr, Rg, and Rb are reflectance values for the red, green, and blue bands respectively [44]. The reflectance values in both the visible and near-infrared (NIR) wavelength bands were used to estimate vegetation health, as was the NDVI. Figure 13 shows the generated result of NDVI for the communities of both Hessyan and Jabal Ali.

#### 2.4.4 Data evaluation (QA/QC)

To evaluate the results of vegetation cover extraction from multispectral drone imagery along with plant disease and pest detection across the Dubai Emirate, the following measures were identified: omission called false negatives and commission errors called false positives, detection rate, and accuracy index (AI) [45]. The AI that quantifies the trade-off between omission and commission error was calculated as:

(2) AI=100(1−FP+FN)/REF

FP and FN stand for false positives and false negatives, respectively, and REF is the number of reference crops in the study area. Other evaluation indices were calculated for this study, including Precision, mean Average Precision (mAP), Recall, and the harmonic Mean F1 score. The F1 score is computed using both Precision and Recall. Precision and Recall are defined as:

(3) Precision=TP/(TP+FP)100%

(4) Recall=TP/(TP+FN)100%

In Formula (3) and Formula (4), TP (True Positives) represent true positives that are predicted to be 1 and that actually are 1, which indicates the number of lesions accurately identified by the algorithm. FP (False Positives) represent false positives that are predicted to be 1 and that are, eventually, 0, which indicates the number of lesions inaccurately identified by the algorithm. On the other hand, FN (False Negatives) represent false negatives predicted to be 0 but that are, in reality, 1. False negatives refer to the number of unrecognized lesions. Detection accuracy is assessed the using mAP (Formula (6)). The average accuracy for each category in the dataset is calculated first as described in formula (5).

(5) $$Paverage=\overset{N\;\left(Class\right)}{\underset{j=0}{\sum }}Precision\;\left(j\right).Recall\left(j\right).100\%$$

(6) mAP=Paverage/N(class)

In the formula mentioned above, N (class) represents the number of all categories, Precision(j) and Recall(j) represent the Precision and Recall of class j respectively. The Average accuracy for each category is defined as mAP. The higher the value of the mAP, the greater the recognition accuracy of the algorithm and, conversely, the lower the algorithm's accuracy. On the other hand, the F1 score as one of the most significant indicators, is also introduced to measure the accuracy of the deep learning model. The F1 score takes into consideration both the accuracy and recall of the model as described in Formula (7), thus:

(7) F1=((2∗Precision∗Recall)/(Precision+Recall))∗100%

Frames per second (FPS) is an indicator used to evaluate the recognition speed of the deep learning model. The higher the value of FPS, the faster the algorithm recognition speed and, conversely, the slower the algorithm recognition speed.

## 3 Results

This section presents experiments using actual data corresponding to twelve communities across the Dubai Emirate, covering a total area of 770.75 sq. km. All types of crops were well covered by the selected deep learning models discussed throughout this paper, with an overall accuracy of 85.4%. Furthermore, date palm and ghaf tree detection results were very promising with F1scores of 96.03% and 94.54%, respectively.

### 3.1 Vegetation cover

The results indicate that the deep learning models outperformed the machine learning technique by at least 11% when using only RGB color images, and more than 28% when using NDVI source images. Accordingly, around 43% of the supervised classification results matched the deep learning output features using NDVI, while the matching between the deep learning results and the photo interpretation method reached 96%. The deep learning model using NDVI produced a few more positives than both the deep learning models using RGB and the manual digitization. When verified through field visits, these were, in fact, plants affected by long-term drought that were challenging to classify through photo interpretation. Table 7 summarizes the confusion matrix results generated for the twelve communities as an overall average and provides a summary of the predicted vegetation cover results using both techniques: deep learning (with NDVI and standard RGB) along with machine learning (supervised classification). Where Sensitivity (SN), called also recall (REC) or true positive rate (TPR), reached 0.89 for deep learning using NDVI, which is interestingly suitable for the study area [46]. The best SN is 1.0, whereas the worst is 0.0. On the other hand, specificity (SP) refers to the number of correct negative predictions, also called the true negative rate (TNR). As indicated in the confusion matrix results, the TNR reached 0.95 overall for the deep learning using NDVI, which is relatively high [47].

**Table 7:** *Confusion Matrix Results- Overall Results for the twelve community scope of the study (Average Values)*.

Accuracy criteria	Deep learning using NDVI	Deep learning using RGB	Supervised classification
Sensitivity (Recall)	0.89	0.73	0.54
Specificity	0.95	0.82	0.61
Positive Predicted Value	0.9	0.83	0.7
Negative Predicted Value	0.6	0.5	0.3
Prevalence	0.7	0.7	0.5
Detection Rate	0.982	0.7	0.6
Detection Prevalence	0.9	0.8	0.4
Balanced Accuracy	0.897	0.7283	0.6154

The vegetation cover was well covered by the land use classification and object-detection deep learning algorithms, with an overall detection and accuracy rate of 89.7% for NDVI and 72.8% for RGB [48]. The commission error was significantly low, the inclusion of bare soil or grass between the crops in the segmentation results was scarce. An individual vector object represented every crop surrounded by around 3–6 cm of grass or bare soil [49]. Table 8 demonstrates the recorded true positives and negatives and false positives and negatives for both deep learning results and the manual digitization layer for the Hessyan community, which shows an overall accuracy index of 87.8%.

**Table 8:** *Results of vegetation cover area as generated by deep learning algorithm using NDVI (Hessyan community)*.

Metric	Deep learning algorithm using NDVI	Reference data (manual digitization)
True Positives	3,520 crops	3,521 crops
False Positives	6 crops	1 crop
False Negatives	12 crops	6 crops
Detection Rate	98.2%	99.9%
Accuracy Index	87.8%	99.9%

Table 9 records the detailed deep learning using NDVI extraction results, including the number of crops, the area covered in square kilometers, and the overall Accuracy Index for the communities selected for the scope of this study. The highest number of crops were recorded in Al Lesaily, Margham, and Remah respectively. These areas also recorded the largest vegetation cover across the Dubai Emirate.

**Table 9:** Results of Deep Learning using NDVI extraction from multispectral drone imagery.

Community	Number of crops	Area sq. km.	Accuracy AI
SAIH SHUAIB	3,117	0.31	97.80%
HADAEQ SHEIKH MOHAMMED BIN RASHID	4,841	1.28	89.70%
ALEYAS	17,435	1.19	86.60%
AL KHEERAN	3,007	1.33	83.90%
AL LESAILY	44,433	2.06	97.40%
MARGHAM	32,379	1.33	85.90%
AL WOHOOSH	4,667	0.03	93.80%
AL MAHA	8,674	0.84	88.90%
REMAH	27,770	0.47	87.50%
GRAYTEESAH	16,049	0.69	84.80%
AL FAGAA	10,298	0.39	92.70%
HESSYAN	3,520	0.60	87.80%
Total/Overall	176,190	10.53	89.73%

**Figure 14:**
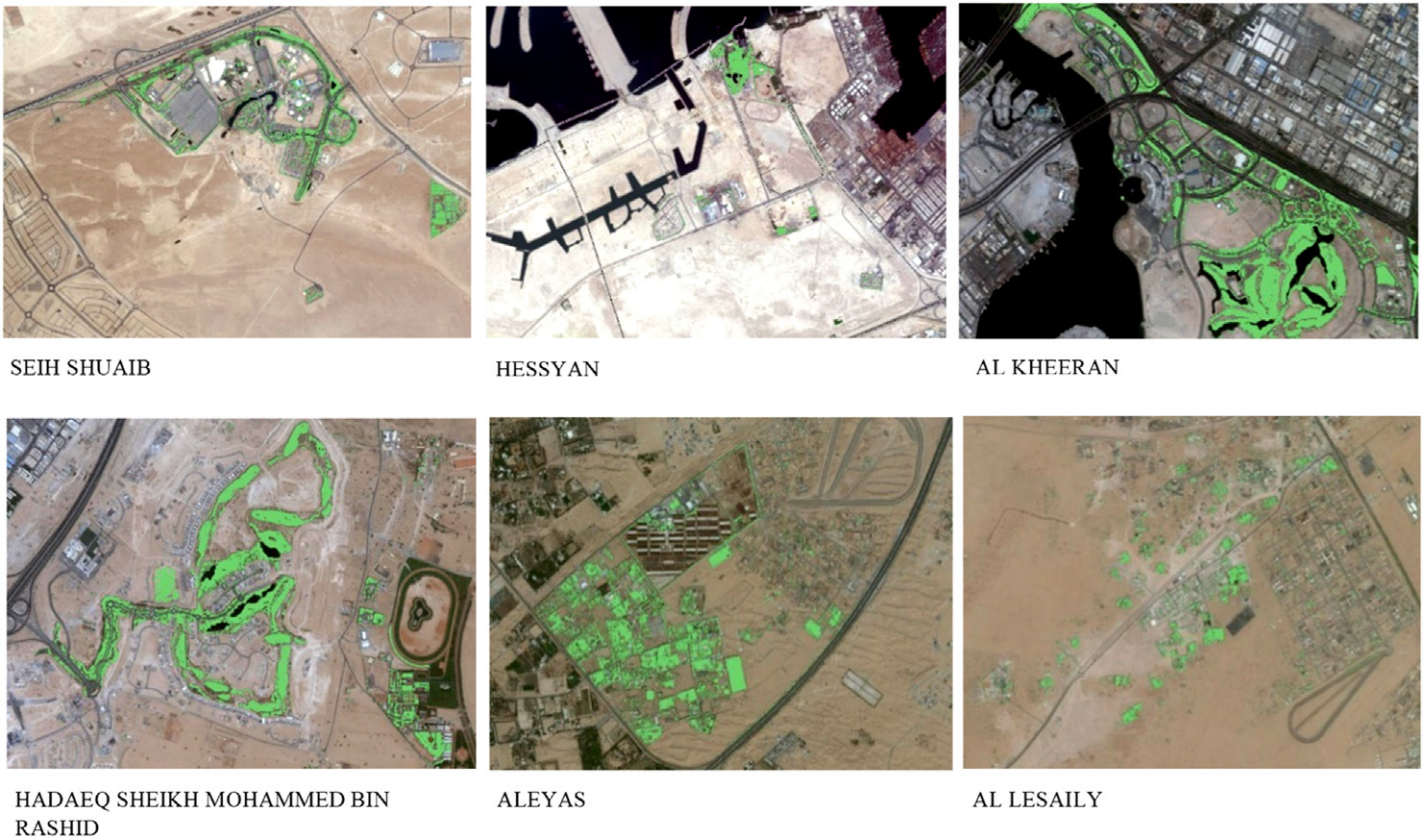
Results of NDVI based deep learning model for vegetation extraction from UAS multispectral imagery.

Figure 14 illustrates examples of the results obtained using the deep learning land use classification model based on NDVI images. As discussed throughout this paper, each source image was carefully prepared and a vector layer representing the vegetation cover was generated by applying a comprehensive methodology that couples deep learning techniques and advanced geospatial analysis. In these results, green color pixels correspond to trees, shrubs, or grass, the silver color corresponds to barren land, while the blue shades correspond to urban land. Interestingly, the highest accuracy is noticed in Seih Shuaib with a 97.8% AI, where several farms utilize hydroponic technology to grow in-demand micro-greens and herbs. In comparison, Al Kheeran has the lowest AI (83.9%). The overall average of the AI calculated for all the communities in the scope of this study is 89.73%.

### 3.2 Detection of date palms and ghaf trees

The accuracy of the tree detection model was evaluated by comparing the results with the actual data derived from the photo interpretation of high resolution drone images. Therefore, this assessment emphasizes counting the number of palm and ghaf trees as the main tree types available across the Dubai Emirate. Table 10 indicates that the deep learning algorithm detects date palm and ghaf trees better when compared with the machine learning method. Once the training phase was successfully completed, the results were tested using a test dataset prepared to be approximately 20% of the training set. The deep learning model predicted 177 date palms and 159 ghaf trees. Thus, this model detected a total of 336 trees on 12 images. The supervised classification algorithm predicted 150 date palms and 139 ghaf trees.

**Table 10:** Comparison of total tree detections by type and method (12 communities).

Metric	Date palms (deep learning)	Date palms (supervised classification)	Ghaf trees (deep learning)	Ghaf trees (supervised classification)
True Positives	10,663	9,238	9,592	7,983
Precision	97.3%	84.3%	95.4%	79.4%
Recall	94.8%	86.8%	93.7%	77.2%
F1 score	96.03%	85.5%	94.54%	78.28%

**Figure 15:**
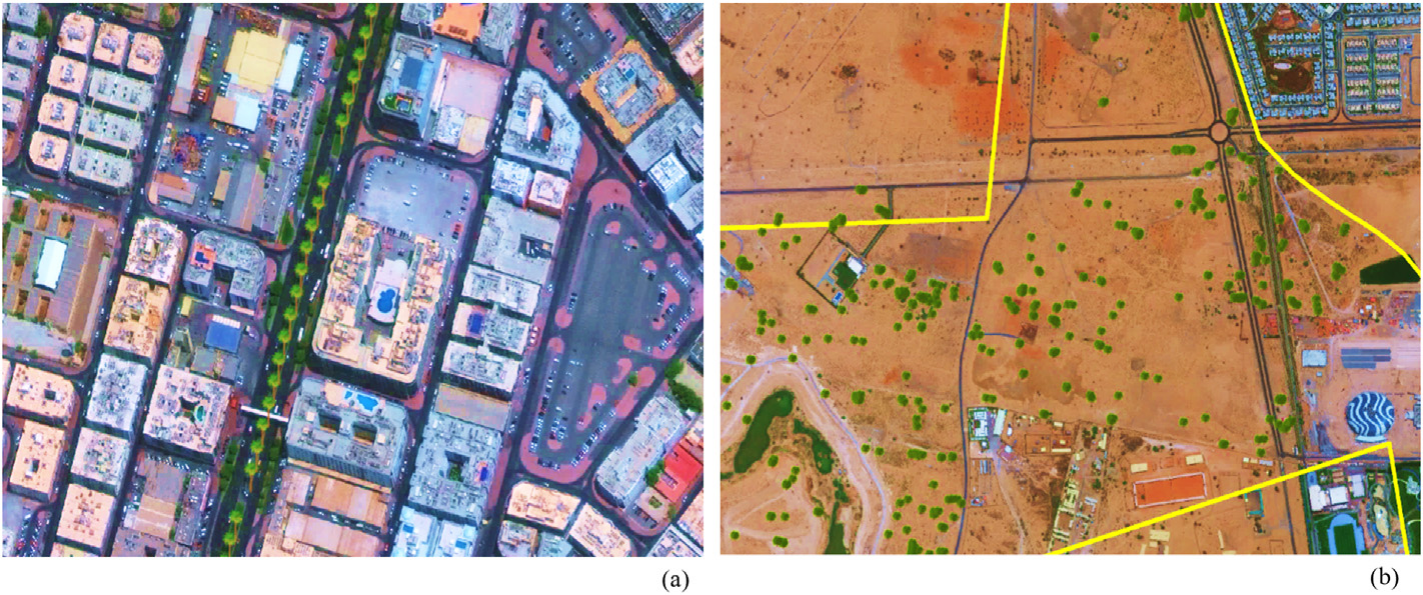
(a): Detected date palm trees along Al KIFAF road; (b): Detected ghaf trees in HADAEQ SHEIKH MOHAMMED BIN RASHID.

In summary, this algorithm detected a total of 289 trees on 12 images. Table 10 illustrates overall results covering 100% of the area of interest [50]. Figure 15 shows an example of the detected date palm and ghaf trees in two different locations. As mentioned in the methodology section, the effectiveness is measured using Precision, Recall, and F1 scores, after comparison with visual interpretation. Each method's performance was evaluated by dividing the prediction results into date palm tree and ghaf tree detection. Overall, the deep learning object detection model showed superior performance when comparing F1 scores. However, the precision percentage for date palm detection is higher than the one calculated for ghaf trees.

**Figure 16:**
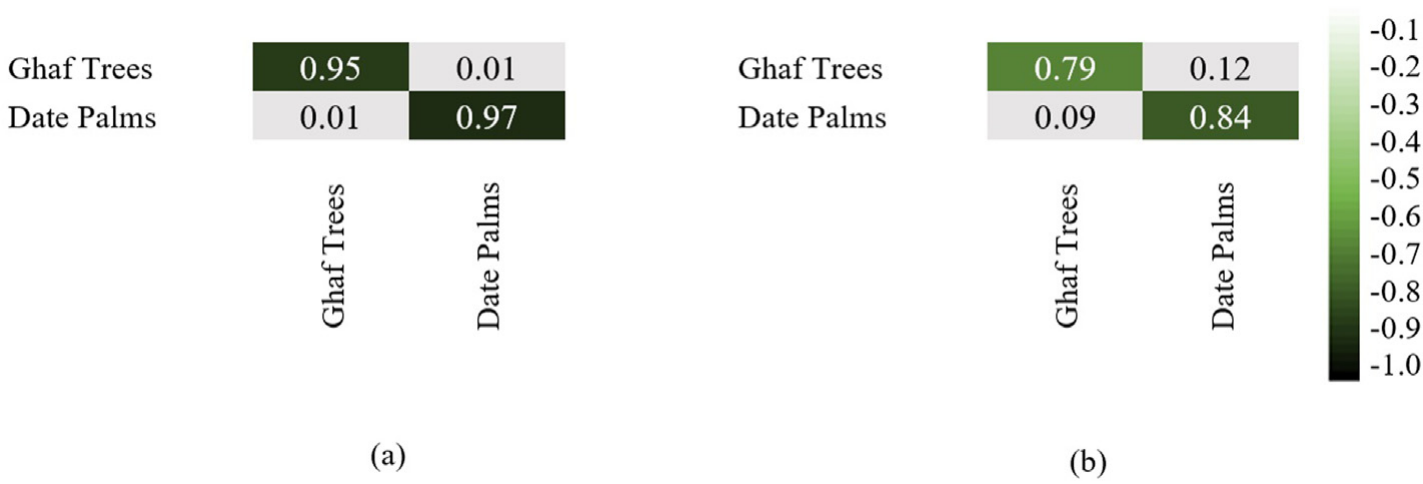
The confusion matrix obtained from different scenarios for both date palm trees and ghaf trees (a) Deep Learning and (b) Supervised classification.

Notably, both methods successfully classified ghaf trees and date palms with relatively acceptable accuracies beyond 79%. However, the deep learning object-based method was the most accurate approach for detecting the target trees with an overall accuracy exceeding 95% and 97%, respectively. Figure 16 demonstrates the differences captured during the detection processes and highlights a higher precision of about 16% for ghafs and 13% for palms.

The initial assessment results indicated that some of the errors occurred due to the trees being obstructed by other tree canopies. Trees with physical characteristics like those of palm trees were another cause of errors. However, these errors were minor and limited to areas where coconut trees are planted. The number of detectable and undetectable palm trees was affected by palms located on the edges of an image, where some parts of the crown area extended across two images. Moreover, the crown size, especially in young palms where it is small, was another cause of detection error. This was due to the small number of young palm samples with small crowns, as the study focused on many mature palms that appear in the target area. However, the errors related to crown size could be addressed to improve performance by increasing the number of young palms in the training data.

### 3.3 Performance comparisons

Tables 7 and 10 summarize the resultant performance metrics of deep learning models using NDVI and RGB imagery versus supervised classification as a conventional way of extracting features in the study area. In the recorded metrics, NDVI deep learning performs better than RGB deep learning. However, the two PyTorch deep learning models perform better than supervised classification (machine learning). This is applicable for both cases of vegetation cover classification and tree object detection. Moreover, the deep learning results with NDVI input channels are found to be slightly better than the deep learning results with RGB input channels only. Among the three methods, object detection using the NDVI deep learning model performs the best. However, in some remote areas like AL WHOHOOCH, REMAH, and AL FAGGA, supervised classification results were satisfactory since the vegetation cover and tree count are not significant in these areas.

Both machine learning and deep learning need enough time to let the algorithms learn and develop enough to fulfill their objectives with an acceptable threshold of accuracy and relevancy. Both also need massive resources to function. This can mean additional computer power requirements. However, machine learning is highly susceptible to errors. Therefore, in some areas, we ended up with biased predictions coming from a biased training set.

Nonetheless, the advantage of the artificial neural networks used by the deep learning models supported this study by providing more accurate results. As mentioned above, the supervised classification offered acceptable results when compared with deep learning in the areas with a smaller vegetation cover ratio. However, whenever this ratio increases, the machine learning accuracy declines significantly. We also noticed that the deep learning models take much more time when compared with machine learning to train a magnitude of a few weeks. The main reason behind this is that many parameters in deep learning algorithms should be identified. In contrast, supervised classification, i.e., machine learning, takes much less time to train, just a few hours. Therefore, we recommend using supervised classification as an alternative to deep learning in situations when the area of interest doesn't have complex or overlapping physical features to save time and unlock high hardware dependency [51].

## 4 Discussion

Dubai is in an arid zone where the desert accounts for more than three-quarters of the Emirate's total area. Its environment is characterized by high temperatures, poor soil, low rainfall, and a lack of natural waterways, which significantly impact the agricultural sector. Although these factors pose significant challenges, the Dubai government has made outstanding efforts over the past few years to build a farming industry capable of contributing to food diversity and the national economy by implementing policies that limit the impact of these factors. In general, the policies are based on innovative technologies and solutions such as hydroponics, aquaponics, and organic agriculture, in addition to strengthening agricultural pest control programs, reducing loss and waste throughout the food chain, and an expanding interest in scientific studies and research in the agricultural field. Table 11 highlights such growth from 2001 to 2019 and demonstrates the efficiency of the policies implemented by the Dubai government to support the agriculture sector in the Emirate. This increase is not a subsidiary event but part of a planned strategy to diversify the economy. The numbers prove this, showing that the average annual growth rate of farms reached 9.3 percent for the past 18 years. While it's not easy to maintain food sustainably in deserts, the agricultural sector in Dubai has witnessed rapid developments in recent years driven by science, innovation, and technology.

**Table 11:** Agriculture lands variation in dūnums from 2001 to 2019.

Year	Agriculture land area in Dūnums	Growth (18 years)
2001	2,602	-
2019	61,914	59,312

In this study, with a 10 cm resolution test dataset, the deep learning model for detecting vegetation cover delivered an overall accuracy of 89.7% for NDVI and 72.8% for RGB. In contrast, the deep learning object detection model produced an average accuracy of 96.03% for date palms and 94.54% for ghaf trees. These results outperform the supervised classification models with an average accuracy of 61.54% for detecting vegetation cover, 85.5% detection rate for date palms, and 78.28% for ghaf trees. Nonetheless, the deep learning method performed considerably better than the investigated machine learning method for vegetation and tree object detection; it needs immense effort to create and maintain training data. Moreover, supervised classification consists of several rules and thresholds that need to be appropriately selected by the user. The parameters and thresholds used in these rules will most likely need to be revisited for another test image beyond Dubai test data. Supervised classification also focuses on vegetation detection as a binary classification problem (vegetation vs. non-vegetation) since it depends on NDVI for detecting candidate vegetation pixels in its first step, whereas there is the flexibility to classify different vegetation types (such as a tree, shrub, and grass) in the deep learning-based methods. Between the two inputs for the deep learning methods, PyTorch provided good detection performance using both RGB images and images with NIR band showing that, for low-budget land cover classification applications, drones with low cost onboard RGB cameras PyTorch deep learning models could certainly be a viable method. Comparing the deep learning using NDVI-based approaches and supervised classification, we observe that the deep learning methods provided significantly better results.

However, one of the considerable limitations of deep learning is that it needs to have a significant number of training images that contain classes to be detected. Such a process might not be practical in cases when time is a project constraint. Our customized deep learning method can handle more than three channels; however, the training was redone from scratch since no pre-trained models were available for the NIR band. One other challenge with deep learning methods is when the dataset is out of balance. With heavily imbalanced datasets, the error from the overrepresented classes contributes much more to the loss value than the error contribution from the underrepresented classes. This makes the deep learning method's loss function biased toward the overrepresented classes resulting in poor classification performance for the underrepresented classes. We didn't encounter this case since Dubai's topography is primarily flat, and the contrast between vegetation and non-vegetation is clear on the images [52].

One of the most critical aspects we carefully attended to during this study was to make sure we created a vast amount of training data. Moreover, the training data needed to have similar characteristics to the testing data. Otherwise, deep learning methods may not have yielded acceptable performance. Augmenting the training dataset using different brightness levels, adding vertically and horizontally flipped versions, shifting, rotating, or adding noisy versions of the training images could be potential strategies to mitigate the issues when test data characteristics differ from the training data.

Taking the results from Table 9 into consideration in this manuscript, the performance of our model is maximal in SAIH SHUAIB (97.8%) followed by AL LESAILY (97.4%), then AL WOHOOSH (93.8%). These are barren areas where living conditions are hostile for both plant and animal life. Consequently, vegetation detection is uncomplicated for our deep learning model since its reflectance is easy to distinguish from other features on the ground. Overall, the advantage of Dubai's nature boosted the performance of our deep learning model in terms of the AI, unlike other countries around the world [53].

The derived maps from the UAV sensors in Dubai using advanced geoprocessing tools coupled with applied deep learning models are currently used in many agricultural applications, including the calculation of the KPIs relevant to the Dubai Urban Plan of 2040 due to their effectiveness in providing high-quality vector datasets and control over the data acquisitions. However, limitations such as the constraint flight time of UAVs and lower coverage make it less affordable than satellite imagery. Therefore, the methodology discussed in this study should also be tested for high resolution satellite imagery to identify if the AI is acceptable for the different purposes where the agriculture and vegetation datasets are used.

## 5 Conclusions

UAS, in combination with deep learning object detection methods, facilitates crop identification and productivity analysis with an overall accuracy of 89.7%, 96.03% detection rate for date palm trees, and 94.54% detection accuracy for ghaf trees. In the Dubai Municipality, such an approach has been proven to have great potential to address and support the most pressing challenges faced by agriculture in terms of access to actionable, up-to-date quality data. Additionally, precision farming combines sensor data and imaging with real-time data analytics to improve farm productivity through the mapping of spatial variability in the field. During this work, the data collected by using drones provided the much-needed wealth of raw data to activate analytical models for agriculture. In supporting precision farming, UAS also help to analyze soil health in the same way that it monitors crop health, assist in planning irrigation schedules, estimate yield data, apply fertilizers, and provide valuable data for weather analysis. Therefore, spatial data collected through drones, combined with other data sources and analytic solutions, provides actionable information. There is a robust correlation between crop yield and the NDVI measured at different crop stages. Hence, tracking the crop growth at critical stages will help provide an accurate estimate of the crop yield and address issues early. The multispectral drone imagery captured during the field missions is one of the best and most effective methods used to detect plants under stress and differentiate between crops and crop stages. Drones fitted with multispectral, infrared, and hyperspectral sensors can accurately analyze soil conditions and crop health. NDVI maps, combined with other indexes such as the Crop-Water Stress Index (CWSI) and Canopy-Chlorophyll Content Index (CCCI) in agricultural mapping tools, provide valuable insights into crop health.

## Declarations

### Author contribution statement

Lala El Hoummaidi: Conceived and designed the experiments; Performed the experiments; Analyzed and interpreted the data; Contributed reagents, materials, analysis tools or data; Wrote the paper.

Abdelkader Larabi; Khan Alam: Contributed reagents, materials, analysis tools or data; Wrote the paper.

### Funding statement

This research did not receive any specific grant from funding agencies in the public, commercial, or not-for-profit sectors.

### Data availability statement

Data will be made available on request.

### Declaration of interests statement

The authors declare no conflict of interest.

### Additional information

No additional information is available for this paper.

## References

[1] Kekane M.A. (2013). Indian agriculture-status, importance and role in Indian economy. Int. J. Agric. Food Sci. Technol..

[2] Fan M., Shen J., Yuan L., Jiang R., Chen X., Davies W.J., Zhang F. (2011). Improving crop productivity and resource use efficiency to ensure food security and environmental quality in China. J. Exp. Bot..

[3] Oyakhilomen R. G. Zibah (2014). Agricultural production and economic growth in Nigeria: implication for rural poverty alleviation. Q. J. Int. Agric..

[4] Awokuse T.O. (2009). The American Agricultural Economics Association Annual Meeting.

[5] Badiene O. (2008). Sustaining and accelerating africa's agricultural growth recovery in the context of changing global food prices. IFPRI Pol Brief.

[6] de Gennaro B.C., Forleo M.B. (2019). Sustainability perspectives in agricultural economics research and policy agenda. Agric. Econ..

[7] Food and Agriculture Organization of the United Nations (2017).

[8] Máté Balogh Jeremiás, Jámbor Attila (2020). The environmental impacts of agricultural trade: a systematic literature review. Sustainability.

[9] Food and Agriculture Organization of the United Nations (2018).

[10] Garsous G. (2019). OECD Trade and Environment Working Papers.

[11] Kwan C., Gribben D., Ayhan B., Bernabe S., Plaza A., Selva M. (2020). Improving land cover classification using extended multi-attribute profiles (EMAP) enhanced color, near infrared, and LiDAR data. Rem. Sens..

[12] Tan K., Zhang Y., Wang X., Chen Y. (2019). Object-based change detection using multiple classifiers and multi-scale uncertainty analysis. Rem. Sens..

[13] Van der Meij B., Kooistra L., Suomalainen J., Barel J., De Deyn G. (2017). Remote sensing of plant trait responses to field-based plant-soil feedback using UAV-based optical sensors. Biogeosciences.

[14] Zare A., Bolton J., Gader P., Schatten M. (2007). Vegetation mapping for landmine detection using long-wave hyperspectral imagery. IEEE Trans. Geosci. Rem. Sens..

[15] Wellmann, T; Lausch, A; Andersson, E; Knapp, S; Cortinovis, C; Jache, J; Scheuer, S; Kremer, P; Mascarenhas, A; Kraemer, R; Haase, A, Schug, F; Haase, D. " Remote sensing in urban planning: contributions towards ecologically sound policies? "; Landsc. Urban Plann., Volume 204, 2020.

[16] Skarlatos D., Vlachos M. (2018). Vegetation removal from UAV derived DSMS, using combination of RGB and NIR imagery. ISPRS Ann Photogram. Rem. Sens. Spatial Inf. Sci..

[17] Hellesen T., Matikainen L. (2013). An object-based approach for mapping shrub and tree cover on grassland habitats by use of LiDAR and CIR orthoimages. Rem. Sens..

[18] Ayhan B., Kwan C., Kwan L., Skarlatos D., Vlachos M. (2020). Proceedings of the Geospatial Informatics X (Conference SI113).

[19] Guirado E., Tabik S., Alcaraz-Segura D., Cabello J., Herrera F. (2017). Deep-learning versus OBIA for scattered shrub detection with Google earth imagery: Ziziphus Lotus as case study. Rem. Sens..

[20] Yang L., Wu X., Praun E., Ma X. (2009). Proceedings of the 17th ACM SIGSPATIAL International Conference on Advances in Geographic Information Systems.

[21] Snehal S.S., Sandeep S.V. (2014). Agricultural crop yield prediction using artificial neural network approach. Int. J. Innovat. Appl. Artif. Intell. Agric. Res. Elect. Elect. Instrum. Control Eng..

[22] Zhang X., Han L., Han L., Zhu L. (2020). How well do deep learning-based methods for land cover classification and object detection perform on high resolution remote sensing imagery?. Rem. Sens..

[23] Song H., He Y. (2005). 3rd International Conference on Information Technology and Applications.

[24] Papageorgiou E.I., Markinos A.T., Gemtos T.A. (2011). Fuzzy cognitive map-based approach for predicting crop production as a basis for decision support system in precision agriculture application. Appl. Soft Comput..

[25] Dai X., Huo Z., Wang H. (2011). Simulation of response of crop yield to soil moisture and salinity with artificial neural network. Field Crop. Res..

[26] Rehman Abdul, Jingdong Luan, Khatoon Rafia, Hussain Imran (2016).

[27] Purkis Sam, Riegl Bernhard (2012).

[28] Bolleter Julian (2019).

[29] Fathelrahman E., Gheblawi M., Muhammad S., Dunn E., Ascough J., Green T. (2017). Optimum returns from greenhouse vegetables under water quality and risk constraints in the United Arab Emirates. Sustainability.

[30] Shahmoradi Javad, Talebi Elaheh, Roghanchi Pedram, Hassanalian Mostafa (2020). A comprehensive review of applications of drone technology in the mining industry. Drones.

[31] Christiansen M.P., Laursen M.S., Jørgensen R.N., Skovsen S., Gislum R. (2017). Designing and testing a UAV mapping system for agricultural field surveying. Sensors.

[32] Starý K., Jelínek Z., Kumhálová J., Chyba J., Balážová K. (2020). Comparing RGB - based vegetation indices from UAV imageries to estimate hops canopy area. Agron. Res..

[33] Klaas Pauly (2016). Proceedings of the 13th International Conference on Precision Agriculture.

[34] Klaas Pauly (2014). 12th International Conference for Precision Agriculture at Sacramento.

[35] Turner D., Lucieer A., Watson C., Turner D., Lucieer A., Watson C. (2012). An automated technique for generating georectified mosaics from ultra-high resolution unmanned aerial vehicle (UAV) imagery, based on structure from motion (SfM) point clouds. Rem. Sens..

[36] Höhle Joachim (2017). Generating topographic map data from classification results. Rem. Sens..

[37] Du Z., Yang J., Ou C., Zhang T. (2019). Smallholder crop area mapped with a semantic segmentation deep learning method. Rem. Sens..

[38] Najafabadi M.M., Villanustre F., Khoshgoftaar T.M. (2015). Deep learning applications and challenges in big data analytics. J. Big Data.

[39] Frank Emmert-Streib, Yang Zhen, Han Feng, Tripathi Shailesh, Matthias Dehmer (2020). An introductory review of deep learning for prediction models with big data. Front. Artif. Intell..

[40] Lamba Harshall (2019).

[41] Michelle Livne, Jana Rieger, Utku Aydin Orhun, Aziz Taha Abdel, Marie Akay Ela, Tabea Kossen, Jan Sobesky, Kelleher John D., Kristian Hildebrand, Frey Dietmar, Madai Vince I. (2019). A U-net deep learning framework for high performance vessel segmentation in patients with cerebrovascular disease. Front. Neurosci..

[42] Gitelson Anatoly, Viña Andrés, Arkebauer Timothy, Rundquist Donald, Keydan Galina, Leavitt Bryan, Keydan G. (2003). Remote estimation of leaf area index and green leaf biomass in maize canopies. Geophys. Res. Lett..

[43] Türkoğlu Muammer, Hanbay Davut (2019). Plant disease and pest detection using deep learning-based features. Turk. J. Electr. Eng. Comput. Sci..

[44] Liu Yu Han (2018). Feature extraction and image recognition with convolutional neural networks. J. Phys. Conf..

[45] Mishra Aditya (2018).

[46] Myagmartseren Purevtseren, Indra Myagmarjav (2018). Cropland suitability assessment and confusion matrix evaluation with GIS. Mong. J. Agric. Sci..

[47] Ohsugi H., Tabuchi H., Enno H., Ishitobi N. (2017). Accuracy of deep learning, a machine-learning technology, using ultra-wide-field fundus ophthalmoscopy for detecting rhegmatogenous retinal detachment. Sci. Rep..

[48] Sogawa T., Tabuchi H., Nagasato D., Masumoto H., Ikuno Y. (2020).

[49] Zhao Hengqian, Yang Chenghai, Guo Wei, Zhang Lifu, Zhang Dongyan (2020). Automatic estimation of crop disease severity levels based on vegetation index normalization. Rem. Sens..

[50] Yarak Kanitta, Witayangkurn Apichon, Kritiyutanont Kunnaree, Arunplod Chomchanok, Shibasaki Ryosuke (2021). Oil palm tree detection and health classification on high-resolution imagery using deep learning.

[51] Wehle Hans-Dieter (2017).

[52] Ayhan Bulent, Kwan Chiman, ree (2012). Shrub, and grass classification using only RGB images. Rem. Sens..

[53] Ayhan Bulent, Kwan Chiman, Budavari Bence, Kwan Liyun, Lu Yan, Perez Daniel, Li Jiang, Skarlatos Dimitrios, Vlachos Marinos (2020). Vegetation detection using deep learning and conventional methods. Rem. Sens..

